# Dynamics of the Presence of Israeli Acute Paralysis Virus in Honey Bee Colonies with Colony Collapse Disorder 

**DOI:** 10.3390/v6052012

**Published:** 2014-05-05

**Authors:** Chunsheng Hou, Hadassah Rivkin, Yossi Slabezki, Nor Chejanovsky

**Affiliations:** 1 Entomology Department, Institute of Plant Protection, The Volcani Center, Bet Dagan, 50250, Israel; E-Mails: houfei2701@hotmail.com (C.H.); dassy@volcani.agri.gov.il (H.R.); 2 Beekeeping Division, Extension Service, Israeli Ministry of Agriculture, Bet Dagan, 50250, Israel; E-Mail: yoslav@shaham.moag.gov.il

**Keywords:** honey bee, colony collapse disorder, Israeli acute paralysis virus, infectivity

## Abstract

The determinants of Colony Collapse Disorder (CCD), a particular case of collapse of honey bee colonies, are still unresolved. Viruses including the Israeli acute paralysis virus (IAPV) were associated with CCD. We found an apiary with colonies showing typical CCD characteristics that bore high loads of IAPV, recovered some colonies from collapse and tested the hypothesis if IAPV was actively replicating in them and infectious to healthy bees. We found that IAPV was the dominant pathogen and it replicated actively in the colonies: viral titers decreased from April to September and increased from September to December. IAPV extracted from infected bees was highly infectious to healthy pupae: they showed several-fold amplification of the viral genome and synthesis of the virion protein VP3. The health of recovered colonies was seriously compromised. Interestingly, a rise of IAPV genomic copies in two colonies coincided with their subsequent collapse. Our results do not imply IAPV as the cause of CCD but indicate that once acquired and induced to replication it acts as an infectious factor that affects the health of the colonies and may determine their survival. This is the first follow up outside the US of CCD-colonies bearing IAPV under natural conditions.

## 1. Introduction

Honey bees (*Apis mellifera*) play a vital role in agriculture by pollinating on a wide variety of crops and flowers. It was estimated that about one third of the agricultural crops in the world depend on honey bee pollination [1]. In the last years there is increasing awareness that honey bee colonies worldwide are suffering a serious decline [2,3]. This decline was attributed to several factors including pesticides, pathogens and parasites such as *Nosema ceranae/ Nosema apis*, *Crithidia mellificae*, *Varroa destructor*, bacteria, as well as viruses [2,4,5,6,7,8,9,10,11]. The most common viruses pathogenic to the honey bee are the Israeli acute paralysis virus (IAPV), The Kashmir bee virus (KBV), the Acute bee paralysis virus (ABPV), the Black queen cell virus (BQCV), the Deformed wing virus (DWV), the Sacbrood virus (SBV) and the Chronic bee paralysis virus (CBPV) [12,13,14,15,16]. Viral infections can remain dormant or undetected in the colony [12,13,14,15,16]. Several studies indicated that stress factors affecting bee immunity may be triggering dormant viral infections remaining hidden within bees or bee colonies, to become overt infections [17,18]. These factors could be human-born such as insecticides [19] or nature-born such as environmental-climatic stresses or co-infection and infestation with other pathogens [17,20].

ABPV, DWV and IAPV were mostly associated with colony losses [9,13,21,22]. ABPV and DWV were correlated with collapse of overwintering colonies in Europe while IAPV was associated with a particular syndrome of colony collapse, labeled Colony Collapse Disorder (CCD), that was observed in California where thousands of colonies are used for almond-tree pollination. These US colonies from migratory operations were found deserted by most of the worker bees, leaving the queen and a small number of bees unable to attend to the abundant brood of the colony, that showed plenty of food and pollen, and no dead bees were found outside of the colony [22]. A metagenomic study suggested a strong association between CCD-colonies and the dicistrovirus IAPV, that was first isolated in Israel [14,22]. Honey bees experimentally infected with IAPV displayed shivering wings and crawling, disorientation, and progressed to paralysis and death within or outside the hive [14,23]. Various IAPV strains were described in the US and other parts of the world [24,25,26,27,28,29,30,31]. Further studies did not directly associate between CCD and the presence of IAPV but indicated that CCD-colonies were prone to pathogen attack, probably due to their exposure to other stress factors [5,6,23,32,33,34,35]. Thus, the phenomenon of disappearing bees received many alternative explanations, but the presence and fate of IAPV in CCD-colonies remained obscure. 

Since the CCD-outbreak in 2005–2007 in the US, there were few reports around the world of colony losses with the characteristics of CCD. Following a call that our Extension Service received about a sudden collapse of 66 colonies in a local beekeeper’s apiary from January to February 2011, we visited the apiary and found a group of colonies displaying classic CCD-symptoms [36,37]. Early-on we detected the presence of significant loads of IAPV in worker bees of these colonies. Given the fact that many viral surveys in colonies around the world pointed to a substantial prevalence of IAPV in covert infections [16,24,25,28,29,36,38], we decided to study the status of this virus in these CCD colonies. We rescued several of these clinical CCD-colonies, avoiding their immediate collapse, to test the hypothesis if IAPV was actively replicating in them and if the virus was infectious to healthy bees. Thus, we studied the occurrence of IAPV over time, its replication and infectivity. Our results indicate that following the initial trigger of infection, IAPV keeps replicating as an infectious factor that can highly affect the health of these colonies. 

## 2. Materials and Methods

### 2.1. Bee Samples

Four honey bee hives (colonies 3, 7, 8 and 10) from a group of *Apis mellifera ligustica* colonies exhibiting symptoms characteristic of CCD in the Ha-Sharon region, Israel, as well as a fifth colony not showing symptoms of CCD but positive for the presence of IAPV (colony 17, for CCD-symptoms see below) and two healthy colonies (16 and 18) from the same location were rescued from the grower’s apiary. These colonies were rescued from of a group of 76 colonies that were looking healthy by January 2012, and one month later 66 of them collapsed with symptoms characteristic of CCD. Namely, most of the worker bees’ population disappeared, leaving the queen and a small number of bees (about 400–800 bees) unable to attend properly to the abundant capped brood of the colony that very rapidly decayed. The colonies showed plenty of food and pollen, and there were no dead bees outside or nearby them. Further, the colonies were not robbed and no wax moths were present. All the above colonies had been fed with sucrose solution before winter, they were regularly monitored and received Fumidyl B and CheckMite (coumaphos) ^TM^ treatment on August 2011. They did not show *Nosema* *apis/cerana* or damaging levels of *Varroa* *destructor* (Varroa levels were checked regularly by the beekeeper one hour after Amitraz-smoking using a white sticky board at the bottom of the colony and there were less than 10 mites per colony). 

The recovered colonies were fed with patties composed of 50% frozen poly floral pollen mixed with and 50% sugar powder (w/w), respectively. Water was added till reaching the right patty consistency. Colonies were fed a 500 g patty once a week.

Sample of 50 adult bees were collected systematically from honey frames that were highly populated and far from the brood chamber at various times during the season. The estimated number of adult bees for a full frame (one side) was 1500 individuals. Brood estimation was based on the calculation of 400 cells per dm^2^. Bee-collection was performed in 50 mL sterile tubes that were frozen immediately at −80 °C until use.

### 2.2. RNA Extraction

Individual bee and larval samples were homogenized in 2 mL sterile tubes with TRI reagent (Sigma-Aldrich) according to the manufacturer’s instructions in a Geno/grinder homogenizer (Metuchen, NJ, USA). Total RNA was dissolved in 20 µL of sterile water and stored at −80 °C until analyzed. Homogenization of groups of 10–30 bees were performed in 15 mL sterile tubes as described above. The quantity and purity of RNA in each sample was measured in a Nanodrop spectrophotometer (Thermo Scientific, Pittsburgh, PA, USA). 

### 2.3. RT-PCR and cDNA

Synthesis of cDNA was performed with 4 µg of RNA samples from above using an oligo (dT)_21_ primer and Maxima reverse transcriptase (Thermo^TM^) according to the manufacturer’s protocol. PCR was performed with forward and reverse primers of the IAPV capsid region: F 5'-GAAGCCCCACTTTGTATGGA-3' and R5'-AGAAACCGCTCCTGAGCATA-3', respectively [19]. For diagnostic PCR amplification of IAPV, we used GoTaq (Promega, Madison, WI, USA). Amplification was undertaken using the GenePro Engine (BioER, Hangzhou, China) with the following thermal cycling profiles: one cycle at 94 °C for 5 min, followed by 34 cycles at 94 °C for 30 s, 55 °C for 30 s, and 72 °C for 1 min, and 72 °C, 10 min. Negative controls were included in each PCR reaction. PCR products were electrophoresed in 1% agarose gel containing 0.5 mg/mL ethidium bromide. IAPV-positive colonies were re-tested with a second pair of primers 

F5'-AGACACCAATCACGGACCTCAC-3' and R5'-AGATTTGTCTGTCTCCCAGTGCACAT-3' to confirm the presence of the virus.

Diagnosis of IAPV, ABPV, BQCV, CBPV, SBV and DWV was performed as described before [36].

### 2.4. Quantitative Real-Time PCR

RNA samples that tested positive for IAPV infection by conventional RT-PCR were subjected to quantitative -real-time RT-PCR (RT-qPCR) using a Rotor-Gene 6000 instrument (Corbett Research, QIAGEN Instruments AG, Hombrechtikon, Switzerland) and forward and reverse primers as described before [24]. Quantitative estimation of positive- *vs.* negative-sense RNA strands were performed by using the tagged primers: 

R5'-agcctgcgcaccgtgACATAGTTGCACGCCAATACGAGAAC-3' and F5'-agcctgcgcaccgtgCCAGCCGTGAAACATGTTCTTACC-3', respectively (lower case tag sequence) to reverse transcribe the extracted RNA and a combination of the above respective primers (without the tag sequence) and the tag primer agcctgcgcaccgtg for the qPCR reaction. The use of this tag sequence non-homologous to *A. mellifera* or honey bee viruses was described before [39].

Housekeeping primers used were RPL8 forward and reverse: F5'-TGGATGTTCAACAGGGTTCATA-3' and R5'-CTGGTGGTGGACGTATTGATAA-3', respectively [40,41]. qPCR was performed with the SYBR Green-based Kapa Sybr^R^ Fast qPCR kit (Kapa Biosystems, Boston, MA, USA) according the manufacturer’s instructions. The cycling profile was 95 °C for 10 min, followed by 40 cycles of 95 °C for 10 s, 60 °C for 15 s, and 72 °C for 20 s. The titers of IAPV were determined by relating the CT values of the measured samples to those of a standard curve prepared by performing qPCR with serial 10-fold dilutions of known concentrations of the IAPV-specific amplicons, about 226 bp in length, and the housekeeping gene to a standard curve of a plasmid carrying the housekeeping gene amplicon as previously reported [37]. DWV was evaluated as described before [41]. Experiments were performed in triplicate for each run. Linear detection over a 7-log range, 10^1^–10^7^ genome equivalents was obtained by plotting CT values *vs.* the logarithm of the concentration of genome equivalent copies. The results were analyzed using Rotor-Gene 6000 Series Software 1.7 (provided by Corbett Research). 

### 2.5. Infection with IAPV

Crude virus inoculum was prepared by crushing ten IAPV-positive adult bees in PBS and filtering it through a 0.2 µm filter. For virus infections, 2 µL of an IAPV crude suspension was injected using Hamilton syringe with a 30 gauge needle into the 3rd and 4th integument of abdomen of white-eyed worker pupae of honey bees. The infected pupae were maintained in an incubator at 35 °C for 3 days. The titer of the inoculated virus was estimated by RT-qPCR from extracted viral RNA.

### 2.6. Protein Extraction and Immunoblotting

Protein was extracted from honey bee samples of CCD- and control-colonies by smashing the bees in PBS containing 0.2% DDC (diethyl dithiocarbamate) and a cocktail of protease inhibitors (Sigma-Aldrich, Rehovot, Israel). After eliminating the tissue debris by centrifugation, the protein extract was washed with chloroform. The aqueous phase containing the soluble proteins was used for analysis. Protein samples were mixed with sample buffer (0.0625 M Tris-HCl, pH 6.8, 2% SDS, 10% glycerol, 5% β‑mercaptoethanol, and 0.125% bromophenol blue) and boiled for 5 min. The polypeptides were separated using 12% SDS-PAGE, transferred to a nitrocellulose membrane and subjected to immunoblot analysis using a polyclonal antiserum that exclusively recognized IAPV VP3 (prepared against a synthetic peptide bearing 20 amino acid residues of VP3, Hylabs, Israel). IAPV protein detection was performed by using an IgG-horseradish peroxidase (HRP)-based conjugate and detection reagents according to the manufacturer’s instructions (ECL, Amersham Biosciences, Pittsburgh, PA, USA) and further exposure to autoradiographic film. 

### 2.7. Statistical Analysis

**Figure 1:**
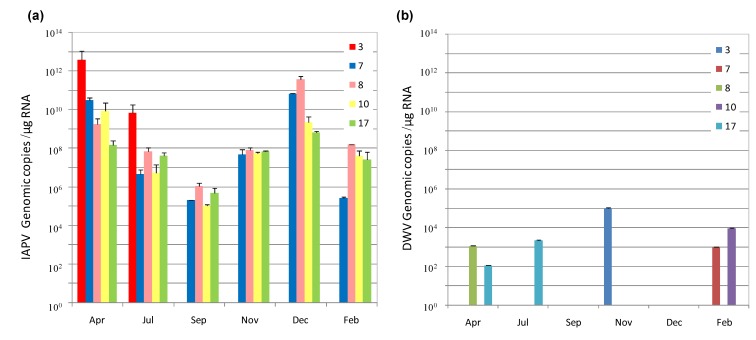
Loads of Israeli Acute Paralysis Virus (IAPV) and Deformed Wing Virus (DWV genomic copies in honey bee samples from CCD- and IAPV- bearing colonies over time. (**a**) IAPV. (**b**) DWV. The viral genomic copy number was determined by RT-qPCR as described in Materials and Methods. Colored bars, different colonies. Each bar represents the average viral load (±SD) of a pool of 10 bees (three replicates). (**a**) The levels of virus varied significantly per colony (colony 3 different from the rest) and per month. (September significantly lower than the rest; April and December significantly higher than the rest) Colony F = 5.8, d.f. = 4,16, *p* < 0.01; Month F = 17.2 d.f. = 4,16, *p* < 0.01, (GLM, Post-hoc test: LSD).

The variation of IAPV titer (triplicates of genomic copies measured from pools of 10 bees) per colony per month was analyzed using repeated measurements (GLM repeated, Stratagraphics 5 plus, 2000). For that purpose, viral titers were log-transformed. The change in IAPV titers was found significant per month and colony (also see legend in Figure 1). 

Differences in positive *vs.* negative-sense RNA strand copies of IAPV were inferred using t-test for all tested pairs (RNA extracted from 5 individual larvae per colony) [42]. 

Differences in the amplification-fold of the IAPV viral inoculum injected into virus-free larvae (5 individual replicates) was estimated using a dependent t-test [42].

## 3. Results

### 3.1. IAPV Fate in CCD Colonies

We managed to sample 18 colonies from the apiary in the Ha-Sharon region described above for their initial evaluation (Table 1, columns 1 and 2). Qualitative RT-PCR analysis of samples from these colonies indicated the presence of several honey bee viruses, mainly IAPV and DWV (Table 1). To estimate the IAPV titers in the colonies we performed quantitative determinations of its genomic copies using RT-qPCR. This test showed that IAPV was the prominent virus in CCD-colonies sampled in March 2011 with genomic copy titers varying from 10^10^–10^12^/µg RNA in colonies 1–15. Colony 17 that did not show CCD-symptoms, showed titers of IAPV below 10^5^/µg RNA, and two colonies, 16 and 18, did not show IAPV at all. Since RT-PCR detected also DWV, an infectious virus frequently associated with colony collapse, we measured its titers in all the colonies by RT-qPCR and found that they were below 10^4^ copies/µg RNA.

From the surviving colonies we managed to follow four CCD-colonies (colonies 3, 7, 8 and 10) and one colony with low IAPV levels (colony 17) that was not showing evident symptoms of CCD, as well as the control colonies 16 and 18, without IAPV. These colonies were fed as described in Materials and Methods and evaluated qualitatively for the presence of IAPV and other viruses by RT-PCR from April 2011 to February 2012, with a 58–60 days interval between sampling, and followed by quantitative determination for IAPV and DWV as described above. While IAPV was detected in all the samples from CCD-colonies that we examined through the year, DWV was detected with low frequency (Figure 1a,b, respectively). Control colonies 16 and 18 where negative for IAPV and DWV.

There were significant changes in IAPV genomic copies per colony and per tested date. IAPV levels diminished through the season, with titers decreasing from April to September in all the colonies and increasing from September to December (Figure 1a). This seasonal variation confirmed our previous report for IAPV incidence in a country wide survey performed in 2010 [36].

**Table 1:** Viral incidence in colonies exhibiting CCD-like syndrome. Viruses detected in RNA extracted from a pool of 10 adult bees. “+ and −, positive- and negative-virus samples, respectively”. IAPV-positive samples were confirmed with a second pair of primers as described in Material and Methods.

Colony	Symptoms	Virus tested					
		IAPV	SBV	ABPV	BQCV	CBPV	DWV
1	CCD	+	−	−	+	−	+
2	CCD	+	−	−	+	−	+
3	CCD	+	−	−	+	−	+
4	CCD	+	−	−	+	−	+
5	CCD	+	−	−	−	−	+
6	CCD	+	−	−	−	+	+
7	CCD	+	−	+	−	−	+
8	CCD	+	−	−	−	−	+
9	CCD	+	−	−	+	−	+
10	CCD	+	−	−	+	−	+
11	CCD	+	−	−	−	−	−
12	CCD	+	−	−	−	−	+
13	CCD	+	−	−	−	−	+
16	Control	−	−	−	+	−	+
17	Non-CCD	+	−	−	−	−	+
18	Control	−	−	−	−	−	−

From all the colonies, colony 3 showed the higher number of IAPV genomic copies in April and in July it was still showing the highest titer of IAPV (over 10^12^ and about 10^10^ genomic copies per µg RNA, respectively). It collapsed by the end of July. Colony 8 reached a peak above of 10^11^ genomic copies per µg RNA in December and collapsed by the end of February. Colony 17 that firstly showed low IAPV titers and no obvious symptoms of CCD experienced an increase in IAPV genomic copies by April and this virus’ levels fluctuated as in the CCD-colonies (Figure 1a). 

### 3.2. Replication and Infectivity of IAPV in CCD-Colonies

The data from Figure 1a suggested that IAPV was replicating actively in IAPV-positive colonies. To directly address this question we tested for IAPV-replication and infectivity with samples of these colonies by three different means:

1. By measuring the relative levels of positive- *vs.* negative-sense RNA strands of IAPV present in individual adult bees of these colonies, a common criterion used for evaluation of replicating positive-strand RNA viruses [37].
2. By injecting virus-free pupae from healthy colonies with extracts of virus from infected bees from the above colonies and measuring if IAPV titers increased significantly following the infection.
3. By injecting virus-free pupae from healthy colonies with extracts of virus from infected bees from the above colonies and following the expression of the viral capsid protein VP3 due to the infection.

**Figure 2:**
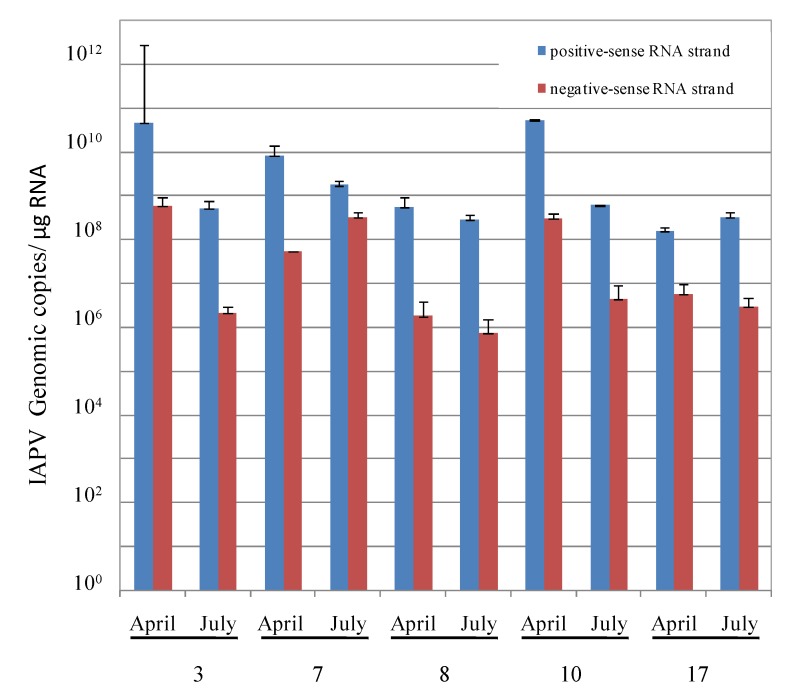
IAPV replication in honey bee samples from CCD-colonies. Number of copies of positive- and negative-sense RNA strands measured by RT-qPCR as described in Materials and Methods. Sample dates and colonies are indicated at the x-axis. All tested pairs +/− were significantly different (t-test, *p* < 0.05).

To detect IAPV replication, we reverse transcribed and amplified both the existing positive-sense strand and the putative negative-sense RNAs of IAPV from bees collected in April and July and quantified them by real-time PCR (see Materials and Methods). We detected positive and negative-sense RNA strands in all the samples tested (Figure 2). Further, the positive- to negative-strand RNA ratios observed were about 100-fold, indicative of IAPV replication (Figure 2, ibid.)

**Figure 3:**
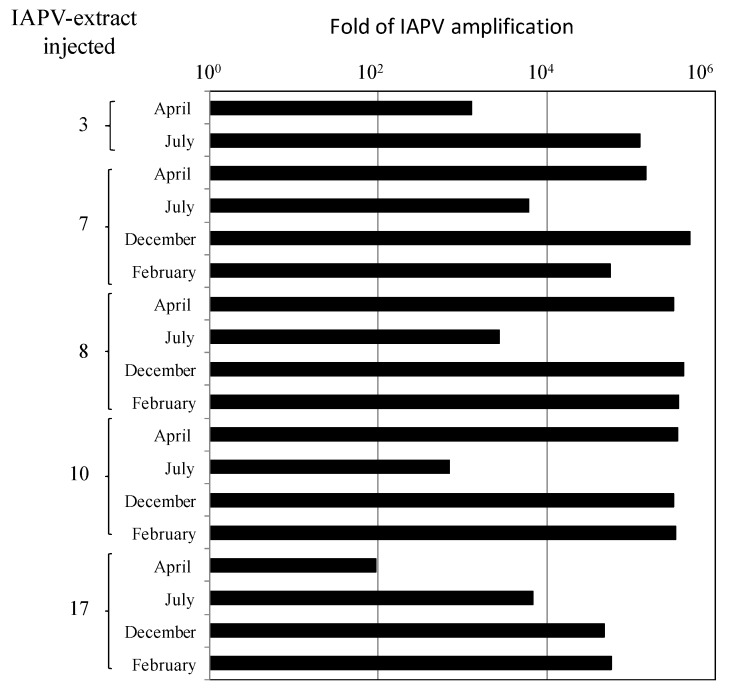
Infectivity of IAPV extracted from CCD-colonies. Virus-free pupae (white-eye) from a healthy colony were inoculated with IAPV-extracted from naturally infected bees from CCD- and 17-colonies. X-axis, amplification-fold of IAPV genomic copies at 72 h after injection of IAPV-extracted at various time points in the year, y-axis. (Dependent t-test, *t* = 3.27215, *p* < 0.01).

To investigate if IAPV from the above colonies was infectious, we injected virus-free pupae from healthy colonies (from a colony pre-tested for the absence of IAPV, ABPV, DWV, SBV and CBPV) with extracts of virus from IAPV-infected bees. We found that in all cases the injected IAPV genomic copies were amplified by several fold of magnitude from the original viral inocula (Figure 3). Larvae injected with extracts prepared from IAPV-free bees were negative for IAPV in RT-PCR tests (not shown).

**Figure 4:**
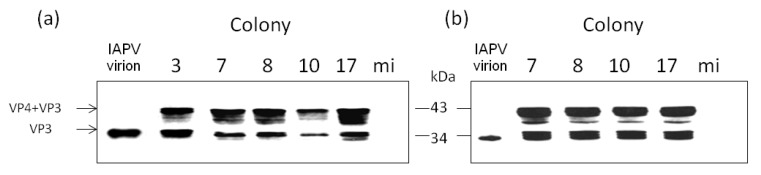
IAPV-virion proteins are synthesized in larvae injected with IAPV extracts of bees from CCD-colonies. Immunoblot analysis of protein extracted from virus-free larvae injected with IAPV extracts from colony samples (indicated above the figure). Panels (**a**) and (**b**), injection of IAPV samples from July 2011 and February 2012, respectively. All viral samples were separated by PAGE and immunoblotted with anti-VP3 antiserum. Arrows, virion proteins recognized by the serum. Molecular size markers are indicated on the right. mi, control mock-injected larvae with extracts derived from healthy bees.

Immunoblot analysis using an anti-VP3 IAPV antiserum of pupae injected with IAPV samples from bee adults of the colonies in Figure 1 (see Materials and Methods) showed that the VP3 capsid protein of IAPV was expressed in the infected bees. Thus, pupae injected with IAPV extracted from samples from July, December and February bees of the colonies 3, 7, 8, 10 and 17 not only amplified the viral genome but also were able to synthesize the virion protein 3 of about 33kDa (Figure 4, panels a and b, only shown for samples from July and February, respectively). The viral protein was not present in mock-injected larvae (Figure 4, ibid.).

### 3.3. Honey Bee Population in Recovered CCD-Colonies

**Figure 5:**
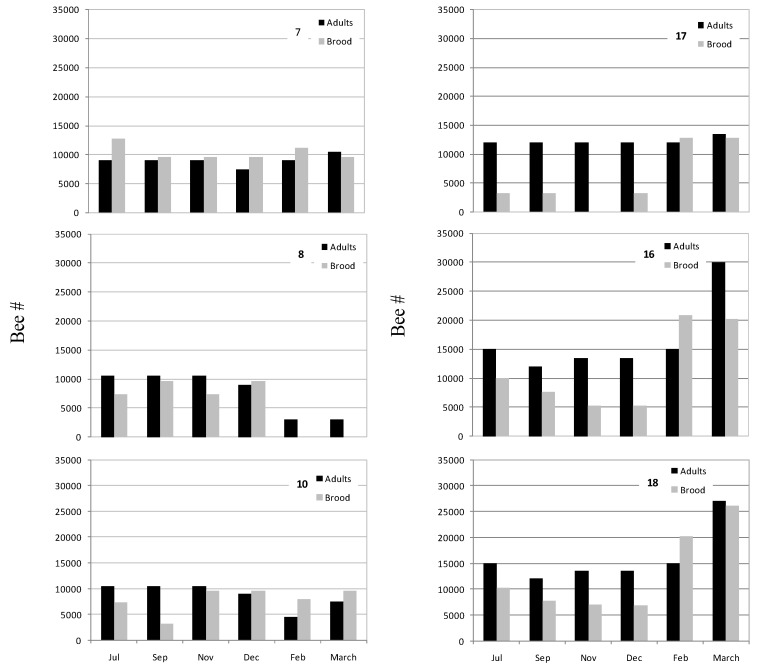
Honey bee population in IAPV-positive, CCD- and healthy control colonies. Adult (black) and brood (gray)**.** CCD-colonies, 7, 8 and 10. Non-CCD colony, 17. Control colonies, 16 and 18. Colony numbers are indicated in each plot.

We were able to follow the CCD-colonies recovered and monitor their brood and adult population as compared to normal colonies, with the exception of colony 3 that collapsed in July. Overall, most of these colonies showed a smaller adult population compared with the control colonies 16 and 18 (Figure 5). Colonies 7, 8 and 10 had reached an adult population of about 10,000 bees from July to November 2011; colony 17’s adult population was about 12,000 bees. Colonies 16 and 18 showed about 15,000 bees in July and 13,500 in November. The adult population of colony 8 decreased from December to February, it was depleted from brood in February and collapsed in March. The adult population in of colonies 7 and 10 showed a mild increase from February to March as compared to the population in control colonies 16 and 18 in the same period. Their brood level was 11,200 and 8000 in February, and 9200 in March, respectively, in contrast to the robust increase observed in the above control colonies (Figure 5). Colony 17 that was infected with IAPV was depleted of brood in November but new brood was observed in December after re-queening. 

## 4. Discussion

After four years of research, we were surprised to find colonies with typical CCD-symptoms in a spotted region of Israel. We noticed that IAPV was the dominant pathogen observed in these colonies, a situation that reminded us of the first study performed in the US following the discovery of CCD [22], the large-scale collapse of managed honey bee colonies during the winter of 2006/2007 that drove the attention of the world to a larger phenomenon: the decline of honey bee colonies. The cases of colony losses described in Europe and elsewhere did not match precisely the symptoms that characterized CCD [9,43,44,45]. Reports that followed from Spain recapitulated only some of the CCD features. Moreover, besides one isolated case in Switzerland, no other typical CCD-cases were reported elsewhere [46]. In the Spanish case, the losses were attributed to *Nosema ceranae* (Microsporidia), and in Switzerland, the researchers did not find a dominant pathogen that correlated with the phenotype observed [46,47]. 

These studies were followed by other investigations that inferred that pathogens and not necessarily IAPV may ultimately cause this particular pattern of colony collapse ([5,22,34] and see below). Still, since many viral surveys in colonies around the world detected a substantial prevalence of IAPV in covert infections [16,24,25,28,30,36,38] we decided to study the status of this virus in the CCD colonies that we recovered. 

We found that IAPV was the prevalent and most dominant pathogen in these colonies with a high number of genomic copies (10^10^–10^12^ copies/µg RNA). Also, IAPV remained as the dominant virus in the recovered CCD-colonies throughout all the period of study (Figure 1). We observed a drop in IAPV titer from April to September in the colonies (Figure 1, ibid), probably due to the death of infected bees and emergence of new adults, not yet infected or mildly infected. The increase in IAPV copies that was observed from September through December in colonies 7, 8 and 10, suggested a process of re-infection of the adult bee population. Indeed, we confirmed that IAPV was replicating by measuring the ratio of positive- to negative-sense RNA present in colony samples that showed a pattern characteristic of picorna-like viruses [41,48] (Figure 2). Furthermore we confirmed its infectious potential by measuring amplification of the viral genomic copies in healthy pupa that were injected with samples of virus from CCD colonies (Figure 3). In these larvae, we observed also *de novo* production of virion proteins (Figure 4). Taken together, the above experiments indicated that IAPV was actively replicating in all colonies where its presence was detected and it conserved its infectious potential. 

It is conceivable that the continuous infection with IAPV impacted colony health because the size of the adult and brood populations of all the colonies was deeply affected. The structure of the adult *vs.* brood population of the IAPV-infected colonies was very different from that of the control colonies (Figure 5). From December (our winter) to March it looks like there was little or null growth in the overall population of the CCD-colonies as compared to control colonies. The adult population declined in three colonies 3, 8 and 10 (Figure 5, ibid) and two of them collapsed, 3 almost immediately and 8 about one year later. Interestingly, the titers of IAPV in colonies 3 and 8 reached levels above 10^11^ genomic copies/µg RNA before collapse. Scattered bee mortality near the hive entrance was observed before collapse of these colonies but the typical CCD-appearance of abundant brood and reduced adult population was not reproduced. This apparent paradox could be explained by the fact that we were dealing with colonies we recovered from a CCD-event with an inherent weakness and an ongoing infection. However, even this condition was an opportunity to follow the fate of the colony and the status of IAPV-infection under natural conditions that were very difficult to find or reproduce outside of the US. Our data suggest that, in this case, being the dominant pathogen in the colonies, IAPV deeply affected their population. Moreover, a recent study showed that healthy larva injected with IAPV under laboratory conditions exhibited disruption of transcriptional homeostasis, supporting the hypothesis of a critical role of the viruses in colony decline and death [23].

The prominent presence of IAPV in the CCD-colonies that we found could be due to re-activation of a dormant infection by a yet unknown trigger that might enable qualitative change and/or quantitative amplification of IAPV. In this context, it was shown that *Varroa destructor* infestation and neonicotinoid application promoted replication of DWV [17,19,20].

Alternatively, the colonies became infected from outside with a particularly virulent strain of IAPV carried by forager bees in pollen grains where the presence of honey bee virus was documented [49]. At this point, we do not have a definitive answer about it, but we are pursuing in order to elucidate this question. Our study suggests that the high level of IAPV copies reached during a short period of time are related to their collapse; we have observed before that in many other cases, this virus was detected in colonies in the country but the titer measured were substantially lower without having major impact to the colony’s well-being [36,50]. 

## 5. Conclusions

Our case study provided evidence for variation and infectivity of IAPV in CCD-colonies at different seasons under natural conditions. It does not pretend to reflect what happens on a grand scale CCD-event were thousands of colonies collapse. Yet, it provides new information about the characteristics of an active IAPV infection in a CCD-event and paves the way for more extensive studies. We showed that IAPV could replicate in honey bees from CCD-colonies and that it persisted over‑time in the colonies as an infectious agent. Furthermore, the data suggest that, in this case, being the dominant pathogen in the colonies, IAPV deeply affected their population, thus a treatment aimed to reduce the viral levels might be critical to ensure/improve their chances of survival. 
